# The role of replication studies in ecology

**DOI:** 10.1002/ece3.6330

**Published:** 2020-05-22

**Authors:** Hannah Fraser, Ashley Barnett, Timothy H. Parker, Fiona Fidler

**Affiliations:** ^1^ School of BioSciences University of Melbourne Parkville VIC Australia; ^2^ Biology Department Whitman College Walla Walla WA USA; ^3^ School of BioSciences School of Historical and Philosophical Studies University of Melbourne Parkville VIC Australia

**Keywords:** conceptual replication, direct replication, generalizability, open science, repeatability, replicability, reproducibility, transparency

## Abstract

Recent large‐scale projects in other disciplines have shown that results often fail to replicate when studies are repeated. The conditions contributing to this problem are also present in ecology, but there have not been any equivalent replication projects. Here, we survey ecologists' understanding of and opinions about replication studies. The majority of ecologists in our sample considered replication studies to be important (97%), not prevalent enough (91%), worth funding even given limited resources (61%), and suitable for publication in all journals (62%). However, there is a disconnect between this enthusiasm and the prevalence of direct replication studies in the literature which is much lower (0.023%: Kelly 2019) than our participants' median estimate of 10%. This may be explained by the obstacles our participants identified including the difficulty of conducting replication studies and of funding and publishing them. We conclude by offering suggestions for how replications could be better integrated into ecological research.

## Graphical abstract

Repeating (replicating) studies can tell you how valid and generalizable the findings are. Ecologists think that replicating studies is important and valuable but a very small proportion of the ecology and evolutionary biology literature is replicated.

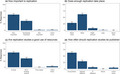

## 1 INTRODUCTION

While replication is often upheld as a cornerstone of scientific methodology, attempts to replicate studies appear rare, at least in some disciplines. Studies looking at the prevalence of self‐identified “replication studies” in the literature find rates of 0.023% in ecology (Kelly, 2019), 0.1% in education (Makel & Plucker, 2014), and 1% in psychology (Makel, Plucker, & Hegarty, 2012). These figures reflect the rate of direct replications where the method from the original study is repeated as closely as possible. Of course, the feasibility of direct replication studies in many areas of ecology is limited by factors such as the challenge of conducting research in originally studied ecosystems which may be remote from potential replicators, the large spatial and temporal scales of many ecological studies, and the dynamic nature of ecosystems (Schnitzer & Carson, 2016; Shavit & Ellison, 2017). However, some subfields, such as behavioral ecology, suffer less from these restrictions and direct (or at least close replications) are more feasible (Nakagawa & Parker, 2015).

In the current study, we are concerned with how researchers think about replication, whether they consider it important, and what epistemic role they believe replication plays in the formulation of scientific evidence.

### 1.1 The role of replication in science

Different kinds of replication studies fulfill different epistemic functions, or purposes. It is common to distinguish between “direct” and “conceptual” replications, where direct replications repeat an original study using methods, instruments, and sampling procedures as close to the original as possible (recognizing that exact replications are largely hypothetical constructs in most disciplines) and conceptual replications make deliberate variations. The dichotomy between direct and conceptual is an oversimplification of a noisy continuum, and many more fine‐grained typologies exist (for a summary see Fidler & Wilcox, 2018) including ecology and evolutionary biology‐specific ones (Kelly, 2006; Nakagawa & Parker, 2015). Broadly speaking, replication studies at the “direct” end of the continuum assess the “conclusion” validity of the original findings (whether the originally observed relationship between measured variables is reliable). Those original findings might be invalid because sampling error led to a misleading result, or because of questionable research practices or even fraud. Replication studies at the “conceptual” end of the continuum test generalizability and robustness, this includes what has previously been termed “quasireplication” where studies are replicated in different species or ecosystems. Where a replication study is placed on the direct‐conceptual continuum and what epistemic function it fulfils depends on the scope of the claim in the original study and how the replication study conforms to or differs from that. For example, imagine I am conducting research in the Great Barrier Reef, and I collect data from some locations in the northern part of the reef. If, after analyzing my results, I make explicit inferences to the Great Barrier Reef as a whole, then studies anywhere along the reef employing the same methods and protocols as the original could reasonably be considered direct replications (within reasonable time constraints, of course). However, if I had constrained my inference to just the northern reef, it would not be reasonable to consider new studies sampling other locations direct replications. Replications beyond the Great Barrier Reef, for instance on coral reefs in the Red Sea, would be conceptual replications in both cases. In Table 1, we illustrate how varying different elements of a study while holding others constant can allow us to interrogate different aspects of its conclusion. However, as the example of the reef demonstrates, whether any given replication is considered direct and conceptual is intrinsically tied to the scope of the inference in the original research claim.

**TABLE 1:** Direct and conceptual replications in ecology. “S” means that the study element in the replication study is similar enough to the original study that it would be considered a fair test of the original hypothesis, and “D” means that the study element is distinctly different in original and replication studies, testing beyond the original hypothesis

	Location	Environmental conditions	Study system	Variables	Epistemic function
*Direct replication*	S	S	S	S	Controls for result being driven by sampling error, QRPs, mistakes, fraud
	D	S	S	S	Controls for result being driven by its specific location within the stated scope of the study
	S	D	S	S	Controls for result depending on the particular environmental conditions at the time of study
*Conceptual replication*	S	S	S	D	Controls for result being an artifact of how the research question was operationalized
	S	S	D	S	Investigates whether the result generalizes to new study systems (often called “quasireplication”)
	S/D	S/D	S/D	S/D	Investigates the generalizability and robustness of the result to multiple simultaneous changes in study design, and potential new interactions

It is worth noting in advance of the next section that the large‐scale replication studies from other disciplines we describe there, and their associated replication success rates, refer exclusively to direct replication studies.

### 1.2 Cause for concern over replication rates

Over the last 8–10 years, concern over a “replication crisis” in science has mounted. The basis of this concern comes from large‐scale direct replication projects in several fields which found low rates of successful replication. Studies included in these projects all attempted fair tests of the original hypothesis, and most were conducted with advice from the original authors. This may mean that the location or time of the replication study differed from the original, but only in cases where location was not specified as being part of the scope of the claim in the original study.

Rates of successful direct replications range from 36% to 62% in psychology, (Camerer et al., 2018; Open Science Collaboration, 2015), from 11% to 49% in preclinical biomedicine (Freedman, Cockburn, & Simcoe, 2015), and from 67% to 78% in economics research (Camerer et al., 2016) depending on the study, and the measure of “successful” used (see Fidler et al., 2017 for a summary).

Low rates of successful replication are usually attributed to poor reliability because of low statistical power in the original studies (Maxwell, Lau, & Howard, 2015); publication bias toward statistically significant results (Fanelli, 2010, 2012; Franco et al., 2014); and the use of questionable research practices (e.g., selectively reporting statistically significant variables, hypothesizing after results known: Agnoli, Wicherts, Veldkamp, Albiero, & Cubelli, 2017; Fraser, Parker, Nakagawa, Barnett, & Fidler, 2018; John, Loewenstein, & Prelec, 2012).

So far, there have been no equivalent, large‐scale replication projects in ecology or related fields. However, meta‐analytic studies have shown that several classic behavioral ecology findings do not reliably replicate (Sánchez‐Tójar et al., 2018; Seguin & Forstmeier, 2012; Wang et al., 2018). In addition, all of the conditions expected to drive low rates of replication mentioned above appear common in ecology and evolution (Fidler et al., 2017; Parker et al., 2016): low power (Jennions & Moller, 2000), publication bias (Cassey, Ewen, Blackburn, & Moller, 2004; Fanelli, 2012; Franco et al., 2014; Jennions & Moller, 2002; Murtaugh, 2002), and prevalence of questionable research practices (Fraser et al., 2018).

### 1.3 Scientists' attitudes toward replication

In the late 1980s, sociologists of science Mulkay and Gilbert interviewed a sample of biochemists about their replication practices. In particular, they were interested in whether these scientists replicated others' work. Most reported that they did not. And yet, the scientists uniformly claimed that their own work had been independently replicated by others. This seems to suggest an implausible state of affairs where everyone's work is replicated but no one is doing replicating (Box 1).

BOX 1 **Excerpt from Mulkay and Gilbert (1991), page 156**

Interviewer: *Does this imply that you don't repeat other people's experiments?*

Respondent: *Never*

Interviewer: *Does anyone repeat yours?*

Respondent: *Oh. Does anybody repeat my experiments? Yes, they do. I have read where people have purified rat liver enzyme from other sources. They get basically the same sub‐unit composition. I'm always happy, by the way which I see that somebody has done something and repeated some of our work, because I always worry…*

Mulkay and Gilbert's explanation of this potential contradiction rested on the notion of “conceptual slippage.” That is, the definition of “replication” that researchers bring to mind when asked about replicating others' work was narrow, centering around direct or exact replication. When considering whether their own work had been replicated by others, they broadened their definition of replication, allowing conceptual replication (different operationalizations and measurements, extensions, etc.). Mulkay and Gilbert referred to the former as “mere replication” and report that it was rarely valued by the scientists in their interview sample. For example, one interviewee referring to another laboratory that is known to replicate studies said: “They actually take pride in the fact they are checking papers that have been published by others, with the result that a great deal of confirmatory work precludes their truly innovative contribution to the literature” (Mulkay & Gilbert, 1991, p. 155).

Dismissal of the value of direct replication research is echoed in Madden's , Easley, and Dunn (1995) survey of 107 social and natural science journal editors, aimed at discovering how journal editors view replication research. Comments from two natural science editors exemplify this “Our attention is focused on avoiding replication! There are so many interesting subjects which have not been studied that it is a stupid thing to make the same work again” and “Why do you want to replicate already published work? If there is some interest [sic] puzzle, of course, but replication for its own sake is never encouraged”. Similarly, Ahadi, Hellas, Ihantola, Korhonen, and Petersen (2016) found a correlation between the perceived value of publishing original research and the perception that replication studies are less valuable in terms of obtaining citations and grant funding.

This negative stigma feeds into the difficulty of publishing replication studies. Ahadi et al. (2016) found that only 10% of computer education researchers that found the same result and 8% that found a different result to the original study were able to publish their replication studies. Baker and Penny (2016) examined the rate of publishing psychology replication studies and found that it was around 12% for replication studies that found the same result and 10% for replication studies that found a different result to the original. This is compounded by the fact that very few people submit replication studies in the first place (Baker & Penny, 2016).

### 1.4 Rationale for the current study

Our goal here is to document and evaluate researchers' self‐reported understanding of, attitudes toward, and (where applicable) objections and obstacles to engaging in replication studies.

The current work investigates Kelly's (2006) argument that there exists in ecology “a general disdain by thesis committees… and journal editors for nonoriginal research” (p232). Echoing findings by Ahadi et al. (2016), Kelly proposed that replication studies may be hard to publish when they agree with the original findings because they do not add anything novel to the literature and also when they disagree with the original findings because the evidence from the original study is given greater weight than the refuting evidence. The current project is, in the broadest sense, an empirical investigation of these issues.

## 2 MATERIALS AND METHODS

### 2.1 Survey participants

We distributed paper and online versions of our anonymous survey (created in Qualtrics Provo, UT, USA, pdf of survey available at https://osf.io/bqc74/) at the Ecological Society of America (ESA) 2017 conference (4,500+ attendees) and EcoTas 2017 (joint conference for the Australian and New Zealand Ecological Societies, 350–450 attendees), in line with ethics approval from the University of Melbourne Human Research Ethics Committee (Ethics ID 1749316.1). We set up a booth in the conference hall at ESA and actively approached passers‐by, asking them to take part in our survey. At EcoTas, we distributed the survey by roaming the conference on foot and announcing the survey in conference sessions. Participants at EcoTas were offered the opportunity to go into the draw and win a piece of artwork representing their research. We promoted the survey on twitter at both conferences. In total, ecologists returned 439 surveys, 218 from ESA, and 221 from EcoTas. Our sample comprises ecologists mostly from Australia, New Zealand, and North America. We have no reason to expect these populations to differ from other populations of ecologists in their opinions regarding replication. However, replication studies in other locations would be needed to assess the generalizability of our results.

### 2.2 Survey instrument

Our survey included multiple‐choice questions about the following:

- How important replication is in ecology
- Whether replication is necessary for the results to be believed or trusted
- Whether there is enough replication taking place
- Whether replication is a good use of resources
- How often replication studies should be published
- Whether participants check for a replication if the study is plausible or implausible
- What types of study do participants consider replications (ranging from computational reproducibility to direct and quasi/conceptual replications)
- We also asked participants to specify the percentage of studies they believe to be replicated in ecology using a slider bar and asked free‐text response questions about following:
- Aside from replications, what might make participants believe or trust a result
- What are the obstacles to replication

### 2.3 Data analysis

The code and data required to computationally reproduce our results and qualitative responses are available from https://osf.io/bqc74/. For each of the multiple‐choice questions, we plotted the proportion (with 95% Confidence Intervals, CIs) of researchers who selected each of the options (e.g., the proportion of researchers who indicated that replication was “Very Important,” “Somewhat Important,” or “Not Important” in ecology) using *ggplot2* (Valero‐Mora, 2015, version 3.2.1) in R (R Development Core Team, 2018, version 3.5.1). All 95% CIs are Wilson Score Intervals calculated in *binom* (Dorai‐Raj, 2014, version 1.1) except for those calculated for the estimate of the prevalence of replication studies in ecology which were generated using parametric assumptions in *Rmisc* (Hope, 2013, version 1.5).

## 3 RESULTS

### 3.1 Prevalence and importance of replication

**FIGURE 1:**
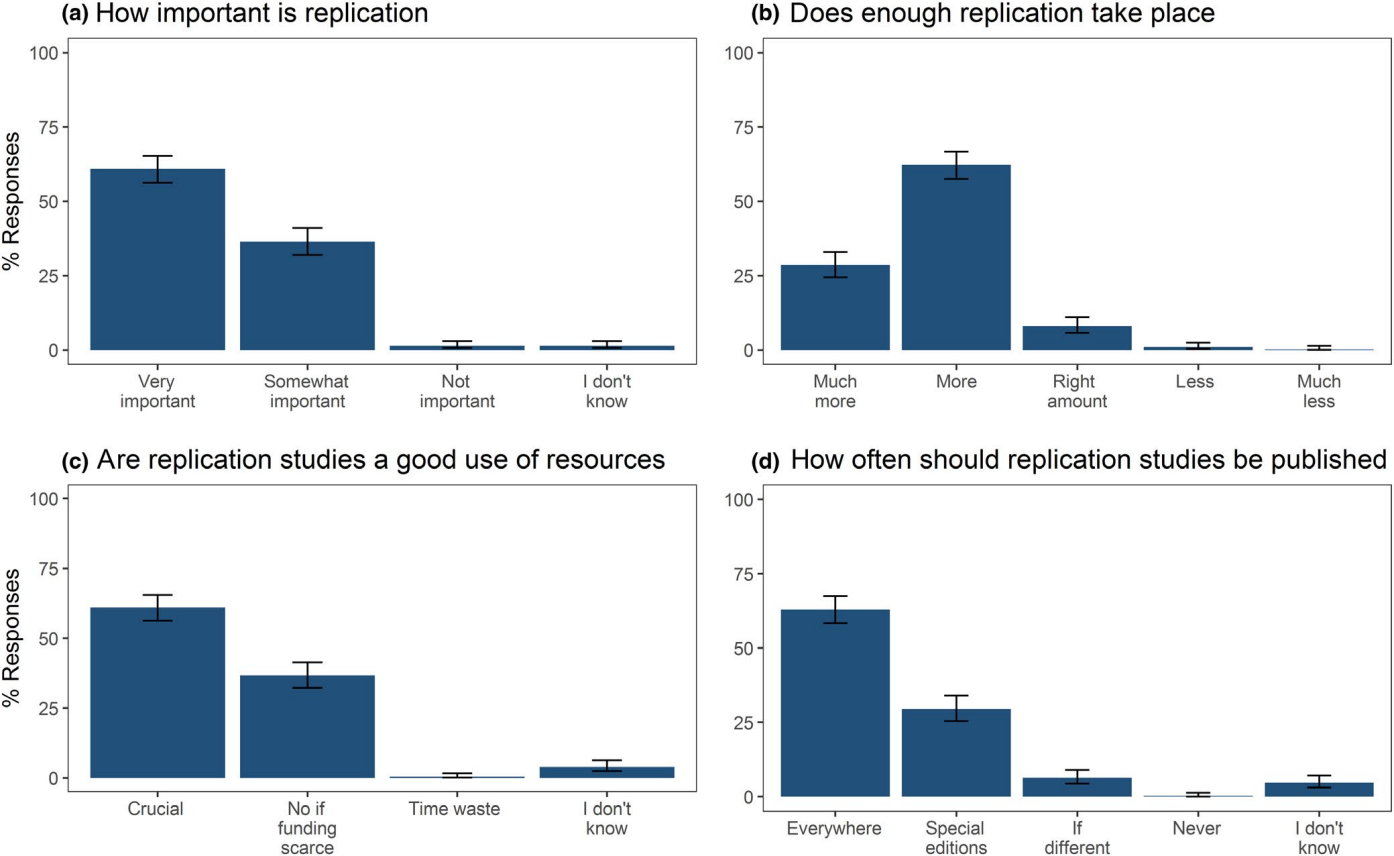
Proportion of participants (with 95%CIs) selecting each option for the following questions: (a) how important is replication in ecology (*n* = 437 participants), (b) does enough replication take place (*n* = 424 participants), (c) do you consider replication studies to be a good use of resources in ecology (*n* = 437 participants), and (d) how often should replication studies be published (*n* = 443 responses from 427 participants)

Our sample of ecologists' median estimate of the proportion of replicated studies was 10% (mean 22%, 95% CIs 20%–24%, *n* = 393). A high proportion of ecologists were very positive about replication. The vast majority (97%, 95%CI: 95%–98%, *n* = 425 of 437 participants) of ecologists answering our survey stated that replication studies are (very or somewhat) important (Figure 1a), and 91% (95% CI: 88%–93%, *n* = 385 of 424 participants) agreed that they would like to see more (or much more) replication taking place in ecology (Figure 1b). Many also agreed that it is “crucial” (61%, 95%CI: 56%–65%, *n* = 261 of 428 participants, Figure 1c) and that replication studies should be published in all journals (63%, 95%CI: 58–67, *n* = 269 of 427 participants, Figure 1d).

Around a third of our sample agreed that replication is important with caveats, suggesting that given limited funding, the focus should remain on novel research (37%, 95%CI: 32%–41%, *n* = 157 of 428 participants, Figure 1c) or that they should only be published in special editions or specific journals (30%, 95%CI: 25%–34%, *n* = 126 of 427 participants). We specifically worded these response items (i.e., pointing to funding scarcity, and publishing only in special issues) to mitigate demand characteristics, that is, undue influence to provide a positive answer to a survey question.

Very few ecologists expressed an overall negative perspective of replication studies; 1% (95%CI: 0.6%–3.0%, *n* = 6 of 437 participants, Figure 1a) agreed that they were not important, 1% (95%CI: 0.5%–2.7%, *n* = 5 of 424 participants, Figure 1b) indicated that there should be “less” or “much less” replication conducted, 0.5% (95%CI: 0.1%–1.7%, *n* = 2 of 428 participants, Figure 1c) agreed that replication studies are a waste of time and money, 6% (95%CI: 4%–9%, *n* = 27 of 427 participants, Figure 1d) indicated that replication studies should only be published if the results differ from those in the original study, and 0.23% indicated that replications should never be published (95%CI: 0.04%–1.3%, *n* = 1 of 427 participants, Figure 1d).

### 3.2 Believability and trust

When asked “does an effect or phenomenon need to be successfully replicated before you believe or trust it,” 43% (95%CI 38%–48%, *n* = 188 of 437 participants) said “yes,” 11% (95%CI: 9%–15%, *n* = 50 of 437 participants) said “no,” and 46% (95%CI: 41%–50%, *n* = 199 of 437 participants) said maybe. This leaves open the question of what participants do use to determine the epistemic value of a finding. Fortunately, 395 (of the total 437) participants provided free text responses when asked what, aside from replication, made an effect or phenomenon more believable or trustworthy (Table 2).

**TABLE 2:** Researchers' (*n* = 395) free‐text responses to a question asking “Is there anything else [aside from replication studies] that you consider to be especially important in determining believability or trustworthiness?” We show summary level results only, with illustrative quotations

	Study design	Open science practices	Reputation	Consistency of current finding with existing knowledge	Statistical qualities of the results
Number of comments	242	68	66	61	53
Indicative quotes	“Sound methodology… appropriate controls, using different approaches/ method to prove the same hypothesis” “Temporal consistency of relationships. Test of consistency across environmental contexts”	“Open, publicly available data and code!” “whether raw data/analysis is presented in published paper supplements or hidden away”	“Sound scientific history of publications. Well regarded in academic or practitioner community” “Reputation of journals (sometimes, but sometimes reputable journals publish crap.)”	“theoretical validity (ie is it biologically supportable through established knowledge or does it severely contradict established theory)” “Are results consistent with similar research? If not, the new research is revolutionary and has a higher bar to convince me”	“degree to which data build the case for the claim (i.e., different approaches (e.g., experimental and observational, different experimental approaches), sites, length of the study) all are useful” “Sample size, power, strength of the effect, how much the findings can be generalised”
Topics covered	‐ scale of the study, ‐ sample size, ‐ use of controls, ‐ statistical approach, ‐ confounds factors	‐ transparent methods, ‐ analysis code available, ‐ data available, ‐ study preregistered	‐ funding source, ‐ conflicts of interest, ‐ reputation of: journal, institution, researcher	consistent with: ‐reader's understanding ‐prior literature ‐existing theory	‐ large effect size, ‐ small p‐value, ‐ result supported by multiple tests, ‐ validity of the data

### 3.3 Checking for replications

**FIGURE 2:**
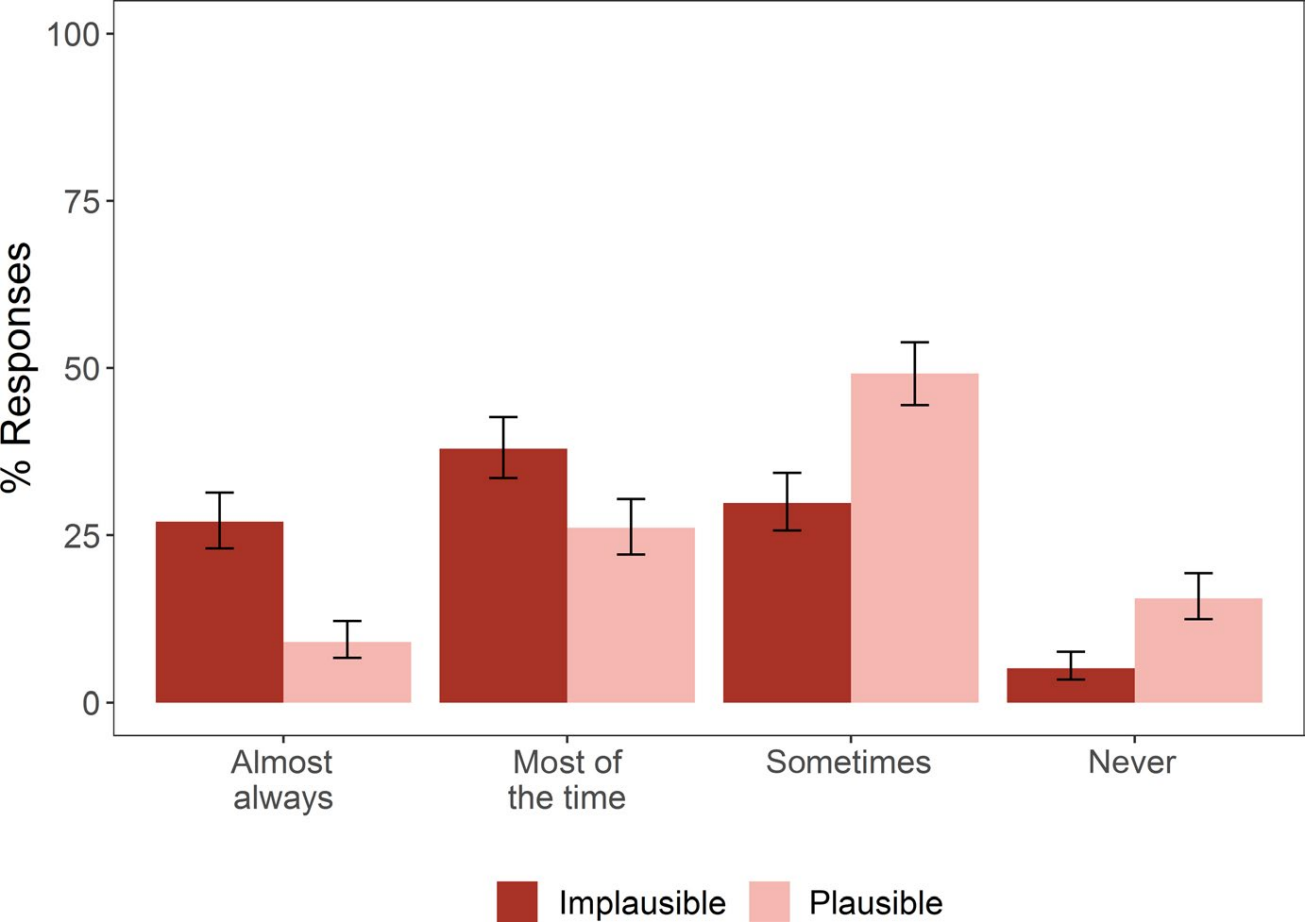
Percentage of participants reporting that they check for replications at different frequencies if the original study seemed plausible versus implausible. Error bars at 95% Wilson confidence intervals (*n* = 429 participants)

We asked how often participants checked for replication studies when they come across an effect or phenomenon that was plausible versus implausible. Very few participants (9%, 95%CI: 7%–12%, *n* = 39 of 429 participants) “almost always” checked whether a study was replicated if they thought the result were plausible. Participants were more likely to check for replication studies if they found the effect implausible but even then, only 27% (23%–31%, *n* = 116 of 429 participants) of participants said that they “almost always” checked (Figure 2).

### 3.4 What is a replication study?

In order to get a picture of what our sampled ecologists consider to be replication studies, we asked participants to select as many options as they wanted from Table 3. The top four options represent the spectrum of replication studies from most direct (first option) to most conceptual (fourth option). The number of participants who considered the options to be replication studies decreased with decreasing similarity between original and replication study. Options 5 and 6 in Table 3 are related to computationally reproducing the results by reanalyzing a study's data. Computational reproducibility is a related concept to replication and has similar, if more limited, epistemic purpose: If the analysis is kept the same, it can detect mistakes and inconsistencies in the original analysis (Table 3, option 5) and, if the analysis is altered, it can test the sensitivity of the findings to alternate modeling decisions (Table 3, option 6).

**TABLE 3:** Statements of different types of variations a new study might make to an original, and the percentage of total participants (*n* = 430) who considered each variation type a “replication study.” Also shown is the mean estimate of the replication rate in ecology, calculated separately for participants who indicated that each of the option constituted a “replication study.”

	Percentage of participants choosing this response (95% CI)	Mean estimate of replication rate in ecology (95% CI)^a^
Redoing an experiment or study as closely as possible to the original (e.g., with same methods and in the context, region, or species)	90% (87–92)	21% (19–24)
Redoing an experiment or study with same (or similar) methods in a new context (region or species, etc.).	73% (69–77)	24% (21–26)
Redoing an experiment or study with different methods in the same context (region or species, etc.).	38% (34–43)	23% (20–27)
Redoing an experiment or study with different methods in a different context (region or species, etc.).	14% (11–18)	19% (13–25)
Re‐analyzing previously collected data with the same statistical methods/models.	41% (37–46)	21% (18–25)
Re‐analyzing previously collected data with the different statistical methods/models.	36% (32–41)	21% (17–24)
None of the above	1% (0–2)	NA

^a^ Mean is used rather than median because it is more sensitive to differences between subsets of participants.

We tested whether different understandings of the definition or scope of replication produced different estimates of the rate of replication studies. We divided participants' estimates of replication rates according to which types of study included in Table 3 each participant considered a type of replication. The estimated replication rate was similar in all subsets.

### 3.5 Obstacles to replication studies

When asked to comment on the obstacles to replication, 407 participants provided free‐text responses, giving insight into why the replication rate might be low (Table 4).

**TABLE 4:** Summary of free‐text responses to the question “In your opinion, what are the main obstacles to replication?”

	Difficulty funding and publishing	Academic culture	Logistical constraints	Environmental variability
Number of comments	332	121	81	36
Indicative quotes	“Given competitive landscape in academia, replication studies hold little reward for researcher‐i.e. no funding/hard to publish/not seen as novel so don't frame you as a research leader in any field” “Hard to publish…very limited resources for biodiversity/ ecology research anyway.”	“I think most scientists want to be known for original work, not for doing ‘some else's’ science.” “Too many things to do, not enough ecologists.” “Lack of emphasis on its importance. funding tends to favour new/novel research. Stigma ‐ people may dislike others who try to replicate their studies. People may consider it ‘lesser or easier science’ replicating.”	“$$ and availability of research sites. When doing field ecology, it can be extremely difficult to replicate sites” “Logistics! Field/ experiments can be expensive and time consuming ‐ also in small populations!” “Hard to find the detailed information necessary for proper replication in original study”	“Long term replication studies are vital to ecology however the problem is climate and habitat loss etc all of which can make it very hard to replicate experiments over time” “Unique attributes of year‐to‐year variability and the challenges that presents ‐ at least for field‐based work for other settings (lab/greenhouse) it seems much more reasonable/worthwhile”
Topics covered	‐ Difficulty funding, ‐ Short duration of funding, ‐ difficulty publishing, ‐ Expect low citation rate, ‐ Not “novel”	‐ Bad for career advancement, ‐ Prioritizing important novel work, ‐ Replications not interesting to do	‐ Not enough time, ‐ Insufficient transparency of methods, ‐ Difficulty accessing original data, ‐ Few candidate sites/populations/individuals	Influence of: ‐ Climate change ‐ Landscape level changes (e.g., caused by clearing or agriculture) ‐ Year on year variation in climate

## 4 DISCUSSION

### 4.1 Importance of replication

The overwhelming majority of the ecologists in our study were very positive about replication studies. They considered replication studies to be important, want to see more of them in the literature and support publishing them (Figure 1a‐d). Enthusiasm for replication studies is further underlined by the sheer quantity of free‐text comments our participants gave (https://osf.io/bqc74). Although we did not give participants a free‐text question about their perspectives on the role of replication studies, some expressed their views about this in the general comments section at the end of the survey. Some evocative examples of these include:

Ecological replication studies should be necessary where results are applied directly to ecosystem management beyond the local/target species context of the study.

Replication means different things in different fields. In biodiversity research replication of studies/phenomena, typically with different settings, species, regions etc., is absolutely essential. The question is when there is enough evidence, i.e. when to stop. There is little point in replicating the study EXACTLY (cf. your question 9 above). In molecular biology or e.g. ecotoxicology it seems that doing the latter actually makes more sense. Different labs should span together and run the same experiment in parallel to eventually publish together.

I think journals should automatically publish replications (or failures to replicate) if they published the original study. I would also be interested in how microbiology vs other biology fields replicate results.

However, there is a disconnect between this message of support for replication studies expressed in portions of our survey and the data on how researchers publish, use, and prioritize replications. First, the best available estimate is that only 0.023% of ecology studies are identified by their authors as replications (Kelly, 2019). This is tiny compared to our participants' median estimate of 10% replication. The disconnect is evident even within our survey, where only a minority of respondents claimed to “almost always” check for replications when investigating a finding (Figure 2), despite emphasizing the importance of replication in other questions and free responses. Similarly, around a third of participants also indicated that, given limited funding, the focus should continue to be on novel research (Figure 1c) and that replication studies should only be published in special editions or dedicated replication journals, or only if the results differ (Figure 1d). This, combined with comments such as “People often want to research something novel, I think there's a mental block among scientists when it comes to replication; most recognize it's necessary, but most aren't particularly interested in doing it themselves,” suggests a gap between the perceived value of replication studies and the impetus to perform them. Comments such as this expose the mistake of assuming replication work—even direct replication—cannot make a novel contribution. For example, working out which aspects of a study are intrinsic to its conclusion and should not be varied in a replication is itself a substantial intellectual contributions (Nosek & Errington, 2017).

This disconnect may be explained by the obstacles identified in this paper, chief among them (a) researchers are, perhaps rightly (Ahadi et al., 2016; Asendorpf & Conner, 2012; Baker & Penny, 2016), concerned that they would have trouble publishing or funding replication studies, (b) conducting replication studies can be logistically problematic, (c) environmental variation makes conducting and interpreting the results of replication studies difficult (Shavit & Ellison, 2017), and (d) researchers are unwilling to conduct replication studies because they believe they are boring and less likely to provide prestige than novel research (Ahadi et al., 2016; Kelly, 2006).

There is movement toward making replication studies more feasible and publishable in other fields, with the inclusion of a criterion describing journals' stance on accepting replication studies as part of the TOP guidelines (Nosek et al., 2015; to which over 5,000 journals are signatories) and the advent of Registered Replication Reports (Simons, Holcombe, & Spellman, 2014) at several psychology journals. Similarly, initiatives like the many laboratories projects (e.g., Klein et al., 2014), StudySwap (https://osf.io/9aj5g/) and the psychological science accelerator (https://psysciacc.org/) build communities that may help overcome the logistical difficulties with replication studies as well as increasing the interest and prestige associated with conducting replication studies. Although no initiatives to directly replicate previously published studies yet exist in ecology, there is a growing movement to improve assessment of generality of hypotheses through collaborations across large numbers of laboratories, implementing identical experiments in different systems (Borer et al., 2014; Knapp et al., 2017; Peters, Loescher, Sanclements, & Havstad, 2014; Verheyen et al., 2016, 2017). The success of these “distributed experiments” suggests that ecologists may be open to forms of collaborations designed to replicate published work.

### 4.2 Conceptual slippage

As in Mulkay and Gilbert (1991), we find evidence of conceptual slippage between different types of replication study. We asked participants whether they consider different types of potential studies “replication studies.” Participants were able to select multiple options. We expected that participants who include conceptual replications in their definition of replication studies would provide higher estimates for the percentage of ecological studies that are replicated. However, there was little difference in participants' estimates of the replication rate regardless of how permissive their definition of replication was (Table 3). This suggests that ecologists have a fluid definition of what a “replication study” is. Similarly, the majority of surveys were distributed by hand, and early in the data collection, it became evident that some were thinking about replicates within a study (i.e., samples) rather than replication of the whole study. As soon as this became evident, we informed each new participant that we were interested in repeating whole studies, not replicates or samples within study. The effect of this confusion on our results is likely to be minimal, because certainly virtually all ecology studies contain within‐study replicates but only 36 of 439 participants (8%) gave answers higher than 50% for the question “What percentage of studies do you think are replicated in ecology?”. This 8% presumably captures all the participants who were answering about “replicates” as well as some that have a very broad definition of what constitutes a replication study.

### 4.3 The continuum of replication

We found very high level of agreement (90%) that “redoing an experiment or study as closely as possible to the original” (i.e., a direct replication) should be considered a replication study. Most ecologists had a view of replication studies that is much broader than direct replication to the extent that 38% considered “redoing an experiment or study with different methods in the same context” and 14% considered “redoing an experiment or study with different methods in a different context” to be replication studies. This permissive definition of a replication study may be driven by the strong influence of environmental variability on the results of ecological research. It is also consistent with Kelly's (2006) observation that conceptual and quasireplication are common in behavioral ecology. Conceptual and quasireplications are required to extend spatial, temporal, and taxonomic generalizability in a field with multitudes of study systems, all of which are strongly influenced by their environment.

Many participating ecologists commented that direct replications may be difficult or impossible in ecology due to the strong influence of environmental variability and need for long‐term studies, concerns that are also voiced by Kelly (2006), Nakagawa and Parker (2015), Kress (2017), and Schnitzer and Carson (2016). Schnitzer and Carson (2016) propose that putting more resources into ensuring that new studies are conducted over a large spatial and temporal scale performing a similar epistemic function as certain types of replication study. Nakagawa and Parker (2015) suggest that the impact of environmental variability can be overcome by conducting multiple robust replications (inevitably in different environmental conditions) and evaluating the overall trends using meta‐analysis. In contrast, Kelly (2006) advocates pairing direct and conceptual replications within a single study, providing insights about both the validity and generalizability of the results and increasing the chance of publication (when compared to a direct replication alone). These suggestions have the potential to make replication studies in ecology more feasible and thereby improve the reliability of the ecology literature. Emphasizing the importance of conceptual replications may also make it easier to build a research culture that is more accepting of replication studies.

Conceptual replications may already be common in ecology and evolutionary biology, but presumably because of the desire to appear novel, such studies are almost never identified as replication. Kelly (2006) found that even though direct replications were absent from a sample of studies in three animal behavior journals, more than a quarter of these studies could be classified as conceptual replications with the same study species, and most of the rest were “quasireplications” in which a previously tested hypothesis was studied in a new taxon. It seems therefore that testing previously tested hypotheses is the norm. We just do not notice because researchers explicitly distinguish their work from previously published research rather than calling attention to the ways in which their studies are replications. In fact, almost none of these conceptual or quasireplications are identified as replications by their authors (Kelly, 2019). This brings up two shortcomings of the current system. First, as pointed out earlier, researchers almost never conduct direct replications, and so the benefits of direct replication in terms of convincing tests of internal validity, are nearly absent. Second, even when researchers conduct conceptual or quasireplications, if they are reluctant to call their work replication, some of the inferential value of their work in testing for generality may be missed. In fact, anecdotally, it seems that inconsistency among conceptual replications is often attributed to biological variation and that this is typically interpreted as meaning that the hypothesized process is more complex or contingent on other factors than originally thought. The generality of the original hypothesis is often not directly challenged.

## 5 CONCLUSION

Most of our participating ecologists agreed that replication studies are important; however, some responses are suggestive of ambivalence toward conducting them. Convincing editors to accept Registered Replication Reports, emphasizing the value of less direct, more conceptual replication, and beginning grassroots replication initiatives (inspired by StudySwap, psychological science accelerator, the many laboratories projects, and existing distributed experiments in ecology) in ecology and related fields may combat ecologists' reluctance to conduct replication studies. Beyond that, we believe that the best approach to replication studies in ecology is to:

1. Identify subsets of studies for which direct or close replication is possible and, because of their importance, value and put resources into such replications. If possible, conduct these as Registered Reports (Nosek & Lakens, 2014).
2. Identify subsets of studies for which direct or close replications are infeasible, and instead put resources into computational reproducibility in those cases. This may include
  - direct computational reproducibility: analyzing the original data using the original analysis scripts (Powers & Hampton, 2019),
  - conceptual computational reproducibility: analyzing the same data with a different analysis method, and/or
  - robustness/sensitivity analysis: analyzing the same data and strategically varying some elements of the analysis as in the multiverse approach (Steegen, Tuerlinckx, Gelman, & Vanpaemel, 2016).
3. Identify subsets of studies for which generalizability is the main concern, and work toward developing “constraints of generality” statements for them (Simons, Shoda, & Lindsay, 2017). Constraints on generality statements explicitly identify the conditions in which the authors think their results are or are not valid. This frees replicators from matching conditions directly and allows replications for generality within constraints laid out by the original authors.

## CONFLICT OF INTEREST

The authors have no conflict of interests.

## AUTHOR CONTRIBUTIONS

**Hannah Fraser:** Data curation (lead); Formal analysis (lead); Investigation (lead); Methodology (supporting); Project administration (equal); Visualization (lead); Writing‐original draft (lead); Writing‐review & editing (lead). **Ashley Barnett:** Conceptualization (equal); Data curation (supporting); Formal analysis (supporting); Methodology (supporting). **Timothy H. Parker:** Conceptualization (equal); Writing‐original draft (supporting); Writing‐review & editing (supporting). **Fiona Fidler:** Conceptualization (equal); Funding acquisition (lead); Investigation (supporting); Methodology (supporting); Project administration (supporting); Resources (lead); Supervision (lead); Writing‐original draft (supporting); Writing‐review & editing (supporting).

### Open Research Badges

This article has been awarded Open Data and Open Materials Badges. All materials and data are publicly accessible via the Open Science Framework at https://doi.org/10.17605/OSF.IO/BQC74.

## Data Availability

All data and analysis code are available at https://osf.io/bqc74/ with a stable https://doi.org/10.17605/OSF.IO/BQC74.
